# Integrating transcriptional activity in genome-scale models of metabolism

**DOI:** 10.1186/s12918-017-0507-0

**Published:** 2017-12-21

**Authors:** Daniel Trejo Banos, Pauline Trébulle, Mohamed Elati

**Affiliations:** 1 UMR 8030 Génomique Métabolique / Laboratoire iSSB CEA-CNRS-UEVE, Genopole campus 1, 5 rue Henri Desbruères, Cedex Évry, 91030 France; 2 Micalis Institute, INRA, AgroParisTech, Université Paris-Saclay, Jouy-en-Josas, 78350 France; 3 Université Lille, CNRS, Centrale Lille, UMR 9189 - CRIStAL - Centre de Recherche en Informatique Signal et Automatique de Lille, Lille, F-59000 France

**Keywords:** Inference and interrogation of regulatory network, Metabolic modeling, *Saccharomyces cerevisiae*

## Abstract

**Background:**

Genome-scale metabolic models provide an opportunity for rational approaches to studies of the different reactions taking place inside the cell. The integration of these models with gene regulatory networks is a hot topic in systems biology. The methods developed to date focus mostly on resolving the metabolic elements and use fairly straightforward approaches to assess the impact of genome expression on the metabolic phenotype.

**Results:**

We present here a method for integrating the reverse engineering of gene regulatory networks into these metabolic models. We applied our method to a high-dimensional gene expression data set to infer a background gene regulatory network. We then compared the resulting phenotype simulations with those obtained by other relevant methods.

**Conclusions:**

Our method outperformed the other approaches tested and was more robust to noise. We also illustrate the utility of this method for studies of a complex biological phenomenon, the diauxic shift in yeast.

## Background

The modeling of biological systems has come a long way for gene regulation, signaling networks and metabolism, but even the most cutting-edge models still focus on one subsystem at the time. The integration of the many subsystems that function together, with the development of modeling paradigms, is the next step in the process, and promising results have already been obtained [1]. For example, [2], constructed a whole-cell model by connecting 28 individual models, one for each of the relevant cell functions. The resulting model included more than 1200 experimentally observed parameters. Impressive as it is, the development of this model required a huge effort for a single organism. We aimed to develop a general methodology that can be adapted to different organisms very easily through minor modifications. We aimed to retain as much information as possible concerning external and internal effects on genotype-phenotype interactions. For example, computational techniques have recently been used to optimize the yield of substrates produced by microorganisms for industry [3] and to study gene-metabolism interactions in medicine [4].

We focus here on the integration between metabolic models and gene regulatory networks for studies of growth phenotypes. Metabolic models represent the chemical reactions required for growth and sustenance [5], whereas gene regulatory networks comprise the biological programs responsible for regulating cell function [6]. We aimed to use data analysis and mathematical modeling tools to improve both the quantity and quality of biological hypotheses relating to these two subsystems.

Related approaches include: pFBA [7] which involves two-level optimization together with post-processing and the detection of redundant fluxes, E-flux [8] in which the linear constraints on fluxes are derived from gene expression data for control and a specific conditions, GIMME [9], which uses gene expression data and a regulatory metabolic objective to detect inconsistencies in fluxes, and iFBA [10], in which a kinetic model of *E. coli* catabolite repression has been integrated into a simplified metabolic model. The iFBA approach requires the setting of a number of Ordinary Differential Equations(ODE) with their kinetic parameters, which decreases the generality of the model. Another integrative approach is PROM [11] in which gene expression data sets are used to compute the conditional probability of an enzyme being expressed given that its regulators are perturbed, these probabilities then being used to constrain a flux balance analysis model. Similarly, TRFBA uses gene expression data and a piece-wise linear model to formulate an optimization program accounting for gene expression [12]. One of the main drawbacks of all these previously described methods is the need to determine which TFs regulate each gene. These approaches are therefore dependent on the quality of the curated network.

We used a statistical reverse engineering method, hLICORN [13], to infer the targets of a given set of regulators at the genome scale. We then assessed the effect of a regulator on its inferred targets in a particular data set, using the CoRegNet [14] tool, which has functions for scoring the activating or repressing effects of a regulator. The derived score, or “influence”, represents the transcriptional state of the cell and forms the basis for posterior integration with metabolic models. CoRegNet allows prior knowledge from various sources to be integrated into the model, in accordance with the recommendations of the DREAM5 consortium [15]. Despite the many and varied publications on gene regulatory network inference [15], few efforts have been made to integrate these inference methods into other systems biology tools.

We based our metabolic analysis on phenotype simulations. We used a well-documented model of yeast metabolism iTO977 [16]. We assembled the inferred gene regulatory and metabolic model together in a rational manner, to simulate growth phenotype and exchange fluxes in an algorithm that we call CoRegFlux. We tested our solution against other state-of-the art methods in a rigorous experimental setting for model benchmarking and comparison [17].

## Methods

**Fig. 1:**
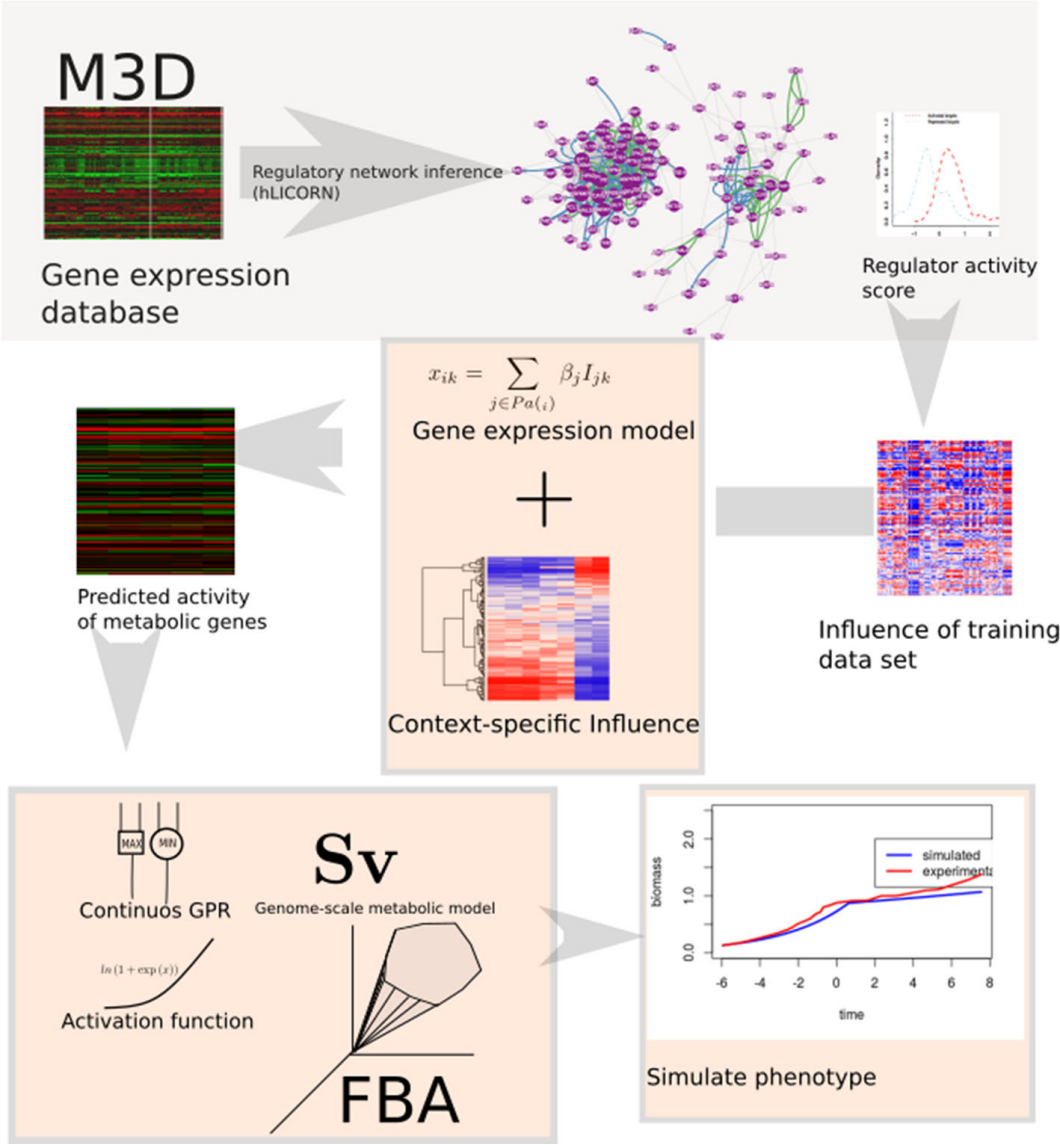
The CoRegFlux workflow, in which a training gene expression data set is used to infer a coregulatory network (in this case from the M3D database [19]). Using this inferred network, we calculated the influence scores of the regulators (i.e. TFs, kinases). We then used these scores to train a linear model for predicting gene expression from influence scores. This model can use context-specific influence scores to predict the activity of metabolic genes in a robust manner. Using these predictions, the model employs a continuous-value version of the Gene-Protein-Reaction rules and a function to translate gene activity into flux bounds. The bounds obtained are then input into a linear program to obtain fluxes congruent with the gene-regulatory state of the cell in a given context

The CoRegFlux workflow can be summarized as follows: inference of the gene regulatory network from transcriptomic data, network interrogation to predict enzyme activity in a given context and, finally, adjustment of the metabolic model for phenotype simulation. The complete workflow is presented in Fig. 1 along with a step-by-step description for a case study in *S. cerevisae* in the sections below, data and source code can be downloaded from http://github.com/i3bionet/CoRegFlux.

### Genetic regulatory network inference

The first step of our algorithm is the inference of a genome-scale regulatory network. This network captures the interactions between regulators (TF and/or kinases) and target genes, which, in our case, encode metabolic enzymes.

For this purpose, we use CoRegNet [14], a Bioconductor package suitable for reverse-engineering and analyisis of large biological networks. Briefly, CoRegNet is a workflow that use the algorithm [13, 18] to mine candidates GRNs set of co-activators and co-inhibitors for each genes. h-LICORN splits genes into regulator and target sets, then discretizes gene expression on the basis of a specified threshold and uses a frequent itemset mining algorithm to find the regulatory elements for each target. In a second step, it determines for each gene the best sets among those candidates by running a regression model. The continuous data can be used alone to refine the original network by selecting for each target the gene regulatory network (GRN) with the best *R* ^2^ score based on the linear model used to estimate the expression. However, CoRegNet can also refine GRNs by incorporating evidence into the network using an integrative selection algorithm and applies it to the selection of local GRN models. Each GRN is scored by the inference method h-LICORN and by each of the integrated dataset. Following this, to each GRN is associated as many scores as they are integrated regulatory and cooperative datasets in addition to the network inference *R* ^2^ score, all which range from 0 to 1. Finally, for each gene, the GRN with the maximum merged score is selected. The refined network obtained is then transformed into a cooperativity network, based on the common targets of regulators.

We began by selecting a data set containing enough gene expression samples to obtain a representative network of gene regulation in yeast: Many Microbe Microarray Database (M3D) [19]. This database contains data from 247 experiments measuring gene expression under different conditions in microarray assays. The data were collated, normalized and averaged (in the case of replicates) for 5520 probes mapping onto ORF.

**Fig. 2:**
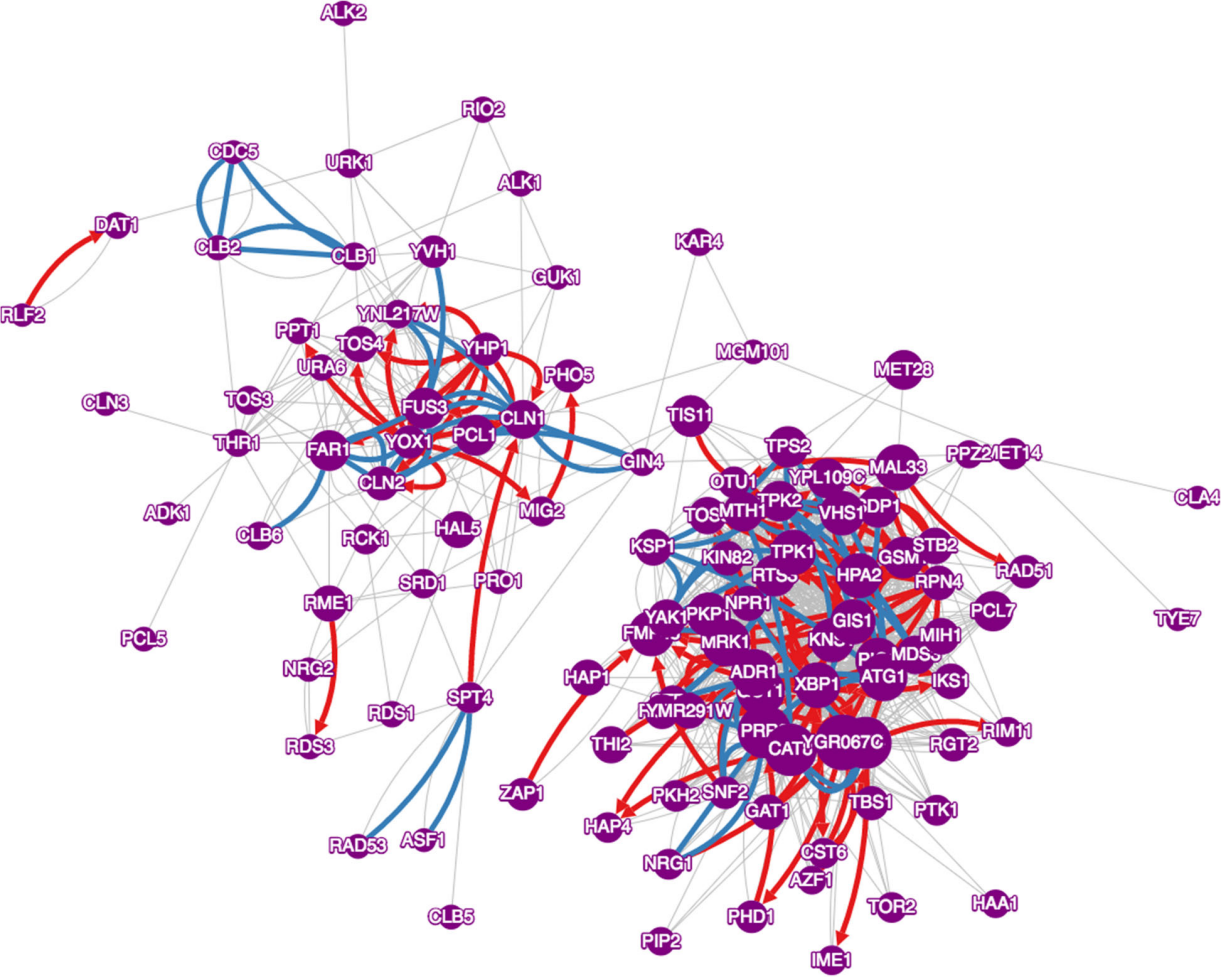
Co-regulatory network representing the M3D data set. Nodes are TF and kinases, grey edges denote co-regulatory interactions discovered by h-Licorn only. Red edges are TF-Target interactions confirmed by Yeastract, blue edges are protein-protein interactions confirmed by Bio-grid

We used CoRegNet to infer a representative regulatory network for yeast. This network should provide insight into the regulators that work together in the performance of a particular biological function. We enriched the network by searching the Yeastract database [20] for known TF-target interactions, and the Biogrid database [21] for known protein-protein interactions. The inferred CoRegNet network has a data structure extending beyond information about regulator-target and regulator-regulator cooperativity [14], see Fig. 2. It also represents the regulatory state of the cell for a given gene expression sample, as explained below.

### Network interrogation and influence score

The bipartite graph generated makes it possible to generate a low-dimensional representation of the transcriptomic data. Nicolle et al. [22] introduced the notion of regulator influence. Here, the impact of a regulator on its targets is represented by the scaled difference of the mean expression levels of its activated and repressed targets. This score is given by the expression

1 $$ I_{j}=\frac{\hat{X}_{A}-\hat{X}_{R}}{\sqrt{\frac{s_{A}^{2}}{n_{A}}+\frac{s_{R}^{2}}{n_{R}}}}  $$

where $\hat {X}_{A}$ and $\hat {X}_{R}$ are the means of the activated and repressed targets of regulator *j* respectively. The variables *s*_*A*_, *s*_*R*_ represent the respective standard deviations and *n*_*A*_, *n*_*R*_ represent the number of genes contained in the respective set. The influence score accounts for the effect of a regulator on its targets according to the regulator-target relationships inferred using hLICORN and additional data-sources integrated in the network as evidences using the CoRegNet Bioconductor package. Briefly, this measure is based on a Welch’t-test between the expression of the activated and repressed targets genes in a given samples.

Thus, a data set of thousands of gene expression measurements is reduced to just dozens or hundreds of activity scores (one score for each regulator with a significant influence). In the case of the M3D data set, the influence score was computed for the set of TFs and kinases as given by [20] (200304 possible TF-target interactions) and the *S. cerevisiae* Kinase and Phosphatase Interactome resource [23] (262354 possible P-P interactions), with a total of 567 potential regulators.

Using this background knowledge of the network and scores, over a wide range of conditions, we aim to predict gene expression and, by extension, the enzymatic activity of proteins encoded, in a metabolic model. We argue that, unlike gene expression alone, influence provides a robust portrait of the transcriptomic state of the cell, improving predictions of the behaviour of targets [22]. We used the inferred network and calculated regulator influences to train a linear regression model over a set of training samples *K*. In this training set the gene expression level of a target in a given sample is a function of the influence of its regulators:

2 $$ x_{ik}=\sum_{j\in Pa\left({~}_{i}\right)}\beta_{j}I_{jk}  $$

where *x*_*ik*_ is the expression level for enzyme *i* in sample *k*, *P* *a*(*x*_*i*_) is the set of regulators of *i* in the network and *I*_*jk*_ is the influence of regulators *j* in sample *k*. The objective of the linear regression is to calculate the regression coefficients *β*_*j*_. For our purposes, we trained the linear model on the M3D data set. Thus, the *beta* coefficients capture the general relationship between gene expression and influence over a wide range of conditions. The linear regression model is then used to predict the level of expression of a gene encoding a given enzyme in the set of context-specific samples of interest. For this, we calculate influence for the samples of interest and predict the gene expression of the metabolic genes with 2. In this study, we used the data set of [24], from a study in which a yeast strain was subjected to various oxygen concentrations. Using the inferred network, we calculated influence scores for this data set. According to the CoRegFlux work flow, we used influence to predict enzyme activity for each sample, based on a linear regression model. We limited predictions to the enzymes present in the genome-scale metabolic model of yeast [16]. These predictions sum up all the available regulatory information for yeast, as given by the inferred network, along with the context specificity of TF influences.

### Metabolic model adjustment

We then translated the predicted enzymatic activities into bounds, corresponding to the extent to which the enzyme encoded by the gene can catalyse a given reaction. These bounds can be used to constrain a linear program representing the metabolic fluxes of a stoichiometric model under the assumption of steady state, a method known as flux balance analysis [25]. The algorithm is as follows:

1. We transform the gene-protein-reaction (gpr) rules, which relate enzyme and enzyme complexes to a given reaction in the model. The original rules are in boolean form and our substitution follows a continuous approximation similar to [26, 27]. Thus the conversion is:
  - OR sentences, which represent isoenzymes regulating the same reaction are subsituted by a function max(), which returns the maximum of the expression of the corresponding enzymes.
  - AND sentences, which represent the formation of enzyme complexes are substituted by a function min() which returns the minimum of the expression of the corresponding enzymes.
  - If the enzyme expression is not available, the enzyme is discarded from the rules.
  We denote the evaluation of a gpr rule for an enzyme-associated reaction by *g* *p* *r*_*r*_(*X*_*pred*_), with *X*_*pred*_ being the set of predicted gene expressions as a function of the influence scores.
2. Using the continuos gpr rules we adjust the fluxes bounds for each gpr-associated flux, denoted *v* ^*r*^ by the following relation
  3 $$ v^{r}\leq\ln\left(1+\exp\left(gpr_{r}\left(X_{pred}\right)+\theta\right)\right)  $$
  where we introduce the parameter *θ* to account for enzymatic action over the reaction. We assume this parameter is condition-specific. We chose the activation function, known as softplus [28], to convey the non-linear relationship between gene expression and protein concentrations. Unlike other non-linear activation functions, like sigmoids, the softplus has a range of (0,+*∞*) making it straightforward to use as flux bounds.

With these new constraints, the flux values and biomass yield can be calculated by solving the linear program associated with the model. We used the R package sybil [29] to find the flux distribution optimizing growth under the new bounds.

### Bayesian optimisation of the parameters

If we wish to adjust parameter *θ* such that the observed phenotype matches the simulated fluxes, a Bayesian optimization algorithm can be used. Bayesian optimization provides an effective out-of-the box solution for a non-linear optimization problem [30]. For the data set of Rintala et al., we ran CoRegFlux, varying the value of parameter *θ* over a uniform grid of 10 points in the interval (-10,10). We then applied the R optimization package [31], to maximize the objective:

$$ \max_{\theta}\left[ -\log \left(\frac{\left\lvert v_{observed}^{B}-v_{simulated}^{B} \right\lvert }{v_{observed}^{B}} \right) \right] $$

**Fig. 3:**
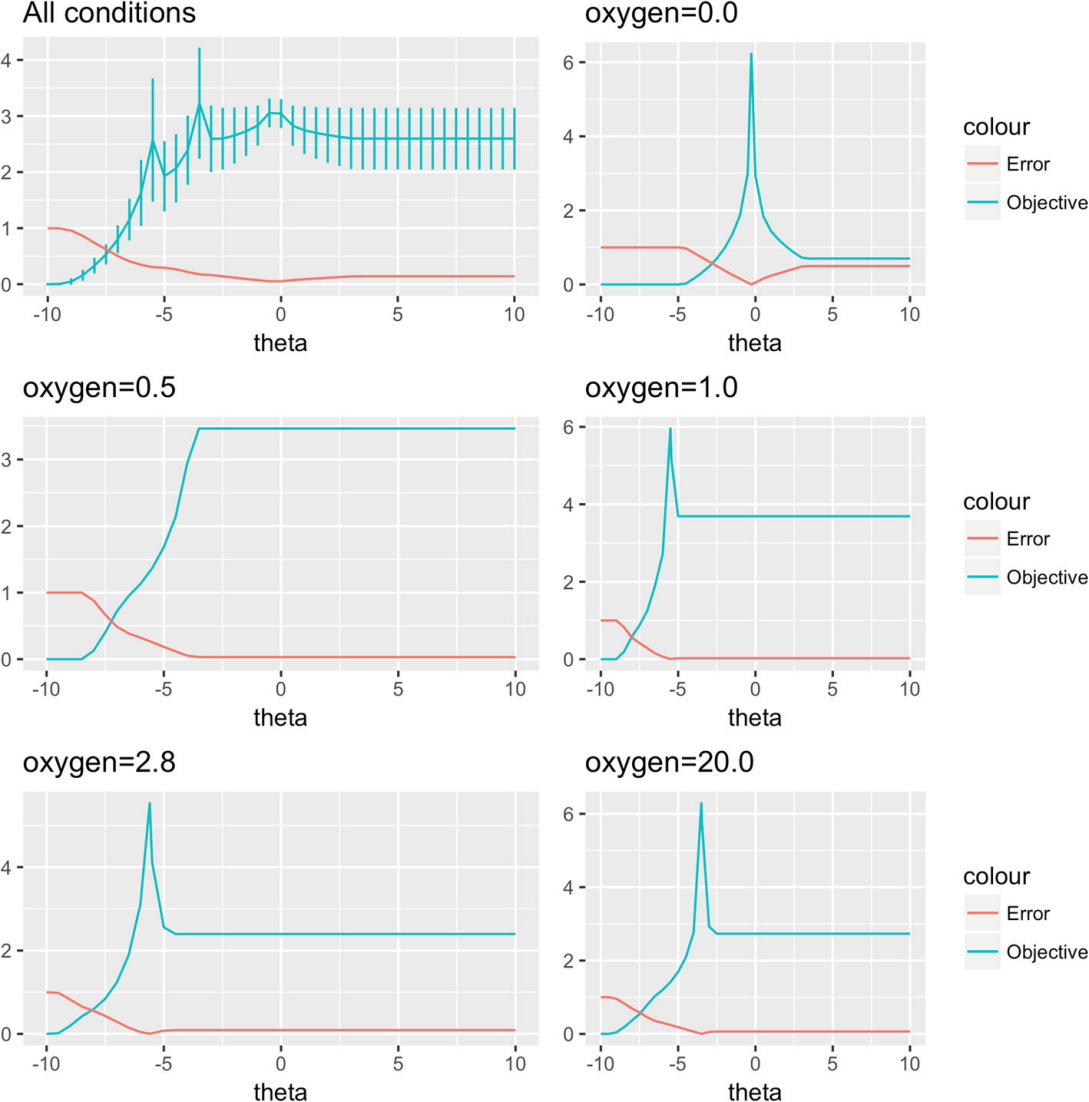
Absolute relative error and objective function for CoRegFlux for different values of theta over all conditions (top left). Values of error and objective function for each condition/oxygenation level (top right, middle left and right, bottom left and right). Bayesian optimization yielded a single optimum for all conditions except an oxygen concentration of 0.5, for which no solution better than the FBA solution was found

This function reaches its maximum when the simulated biomass yield $v_{simulated}^{B}$ is closest to the observed growth rate $v_{observed}^{B}$. We chose to use this function, to improve appreciation of the effect of the parameter value on approximation error. We used the default settings of the Bayesian optimization package [31] to estimate optimal values of *θ* for each condition. The results are shown in Fig. 3, in which, for each condition, the value of the parameter increases as it approaches the optimum value (reducing the relative error). If we continue to increase the parameter, the relative error of the solution settles at the value for the flux balance analysis model. A special case occurs when oxygen concentration is 0.5. In this case, the flux balance analysis solution underestimates the growth yield, and the method is unable to find an optimum value for the parameter. For all other cases, a clear optimum value is identified.

## Results

We evaluated the results generated by our method in terms of the accuracy with which they predicted exchange fluxes and to illustrate the use of this approach in a relevant case study. We performed robustness tests to determine whether influence gave a more reliable picture of the regulatory state of the cell. As our case study we choose the diauxic shift, a complex biological process involving major changes in transcriptional and metabolic elements in yeast.

### Robustness tests

**Fig. 4:**
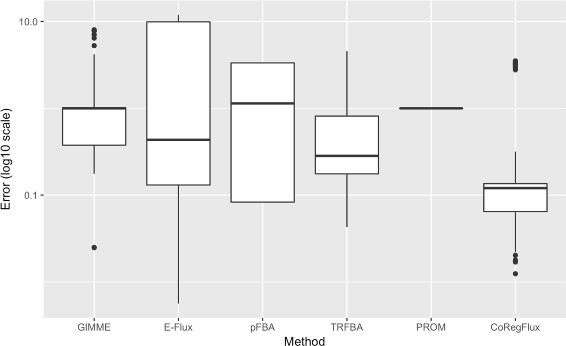
Results for robustness analysis in all conditions and for five different noise levels. The normalized error, corresponding to the difference between observed and simulated exchange fluxes, is shown on the y axis. A log10 transformation was applied to the data to improve readability. CoRegFlux had a lower median error than two other state-of-the-art methods, TRFBA and pFBA

We evaluated the robustness of our method to random permutations of gene expression, as recommended by [17]. We set our *θ* parameter to its optimum value for each condition, and we then tested five different noise levels for each condition. The mean squared error for exchange fluxes was calculated as described by [17]; Fig. 4 shows a boxplot for the base 10 logarithm of the error. We also considered the results generated by competing methods: GIMME, E-Flux, pFBA, TRFBA and PROM [12]. Our method had a better median performance and a smaller variance than the other methods. As all tests were performed in the wild-type strain, PROM [11] displayed no variation, as this method was designed for prediction for knockout strains and does not seem to take regulatory information into account for the wild type.

### Case study

**Fig. 5:**
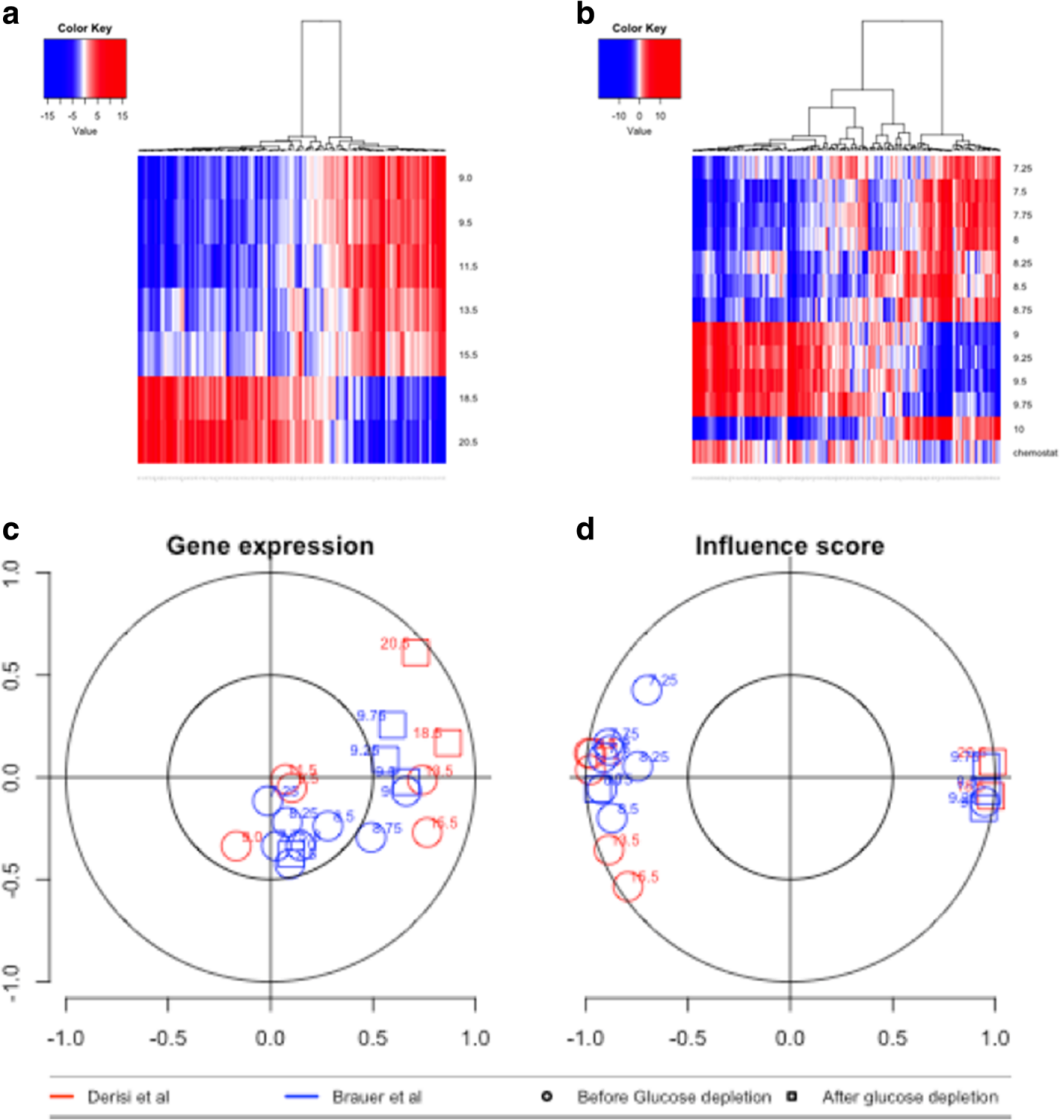
In **a**, heatmap for influences for the data set of Derisi et al., in **b**, heatmap for the dataset of Brauer et al. Positive influences are shown in red and correspond to an upregulation of targets, whereas negative influences are shown in blue and correspond to the downregulation of targets. At **c**, the correlation between samples based on gene expression is shown (calculated by canonical correlation analysis). At **d**, the equivalent correlation plot based on influence scores is shown. Samples obtained before and after glucose depletion can be clearly differentiated on the basis of influence scores, whereas the relationship is less clear for gene expression

We used diauxic shift as a case study, with the gene expression measurements of [32] corresponding to 12 time points (9 before and 3 after glucose depletion). We compared this data set with that of [33], for seven samples during the diauxic shift. We plotted influence score heatmaps for both data sets. We used canonical correlations analysis to compare the correlation between sample points in the two different data sets. We alternated between gene expression and regulatory influence, and the corresponding correlation plot is shown in Fig. 5. Plots based on regulatory influence separated two distinct clusters of samples, corresponding to the samples taken before and after glucose depletion, except for the sample taken at t =9 h [32] appearing closer to a post-depletion state (it should be noted that the authors of the paper reported regulatory changes beginning a few minutes before glucose depletion). Another interesting point is that of t =10 h in [32], for which the influence profile seems more similar to those obtained before glucose depletion, which may point to a growth-state. These interesting patterns were not evident in analyses of regulator gene expression, in which the separation between pre- and post-depletion samples was less clear.

**Fig. 6:**
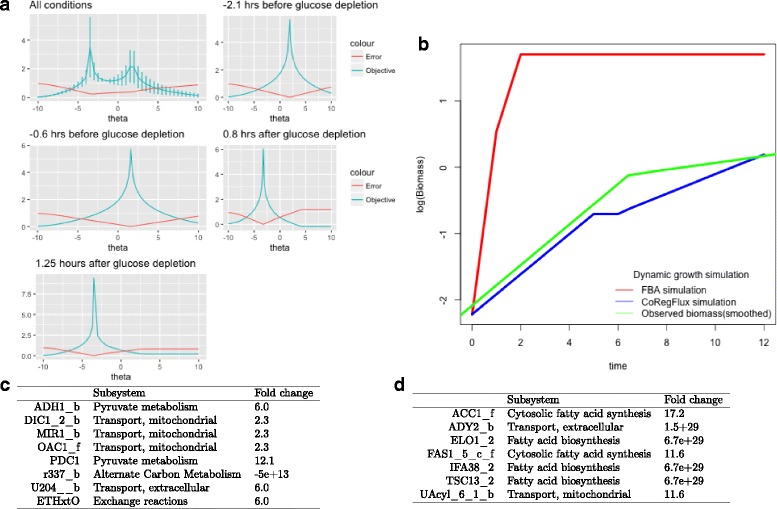
Showed in **a** are the results of calibrating the softplus parameter for the different diauxic phases, two before and two after glucose depletion, the relative errors (red) and objective function (blue) values are plotted. In **b** the log(Biomass) results of a dynamic FBA simulation of growth using a normal FBA model(red) a CoRegFlux model(blue) and comparison with the smoothed growth curve from the experimental data set(green). Table **c** provides a comparison in fold-change between the fluxes obtained by an FBA model and a CoRegFlux model before glucose depletion (t=-0.6 h), positive fold change implies that the CoRegFlux models finds solutions with more mass going through the reaction than FBA, negative values imply reduced flux through the CoRegFlux model compared to FBA. Table **d** is analogous to the previous table but with post-glucose depletion conditions (t=0.8 h)

We matched the sample points to the different regulatory phases identified by [34], with metabolic states attributed to the phases -2.1 and -0.6 h before glucose depletion and 0.8 and 4 h after glucose depletion. In our case, the last gene expression sample from [32] was taken at t =1.25 h after glucose depletion. With these phases in mind, we adjusted the glucose and ethanol exchange bounds to those for the metabolomic data set of [34] and parametrized our CoRegFlux models with the growth rates reported for each phase in [34]. As shown in Fig. 6 top-left, the pre- and post-glucose depletion models had different optimal values for the parameter *θ*, again reflecting the information about the regulatory state of the cell provided by the influence score. We further investigated the differences between CoRegFlux model output and flux balance solutions. The differences are shown in tables bottom-right and bottom left of Fig. 6, in which FBA fluxes and CoRegFlux fluxes are compared in fold change for the pre- exhaustion and post exhaustion respectively, we chose to present only those fluxes that experience more than a two-fold change. The tables show increased ethanol excretion (reaction ETHxt0), in fact ethanol excretion is predicted as 0 by FBA, along with increase transport of metabolites to the mitochondria for the pre-glucose exhaustion phase at -0.6 h which matches the observations of [34]. In the post-glucose depletion state at 0.8 h, CoRegFlux predicts an increase in fluxes regulated by ACC1 and FAS1 genes, which are important in the production of Acyl CoA, which oxydizes and becomes Acetyl-CoA, primary precursor of ATP production by the TCA cycle post shift [35]. Finally we assessed the utility of our model for dynamic growth simulations using a dynamic FBA formulation as in [36]. We used the initial biomass, glucose and ethanol concentrations from [34] and computed the metabolite consumption and growth rate at each time step. We compared the results of using a normal FBA model constrained only to the initial glucose uptake rate (but allowing ethanol consumption), to the results using CoRegFlux models. We proceed as follows: one of the previously constrained models was assigned to the corresponding time points, then at the switch points between diauxic phases, the current biomass and metabolite concentrations was used as initial conditions for the next model. The derived growth curves are presented in top right of Fig. 6, where we see that the FBA model both over-estimates growth and does not initiate the second phase of diauxic growth. The CoRegFlux model on the other hand, follows more closely the smoothed growth curve provided by the authors of said study.

## Discussion and conclusions

We propose CoRegFlux, a new algorithm for integrating gene regulatory network inference with constraint-based metabolic models. Our method provided better median predictions with a lower variance prediction than other state-of-the-art methods for predicting exchange fluxes under different levels of perturbation of gene expression data. One of the limitations of this method is that it cannot determine the optimal parameter value for systems in which the normal FBA solution underestimates biomass yield, although it should be pointed out that FBA overestimates the growth rate in most cases [37]. From the robustness tests and the case study, we can conclude that influence score calculation is a reliable way to assess the overall effects of gene regulation. This advantage of the influence score places it among other approaches to dimensionality reduction for gene expression such as network component analysis [38]. The importance of having a clear idea of the transcriptomic state of the cell has been demonstrated in studies of metabolism and responses to particular conditions. For example, recent results have suggested that at least 70% of the total variance in promoter activity across conditions can be accounted for by global transcriptional regulation in *E. coli* [39].

As mentioned above, this method has potential applications in research, industry and medicine, and its improvement would therefore be worthwhile. For example, it would be interesting to include different models of gene regulation as additional predictors of enzyme activity. Future research studies could also include metabolic network learning, with a view to the development of a data-driven integrated algorithm. Finally, this method is designed to serve as a basis for the *in-silico* optimization of biological objectives, of potential value for experimental design in systems and synthetic biology.
